# Gastroduodenal Obstruction Due to Bouveret's Syndrome: A Case Report

**DOI:** 10.7759/cureus.75175

**Published:** 2024-12-05

**Authors:** Alejandro Osorio-Euan, Victor M Ayuso-Diaz, Jaime A Ferraez-Perez, Angelica Moreno-Enriquez, Grecia F Hurtado-Miranda

**Affiliations:** 1 Surgery, Hospital Regional Elvia Carrillo Puerto, Institute for Social Security and Services for State Workers (ISSSTE) Facultad de Medicina de la Universidad Autónoma de Yucatán, Yucatan, MEX; 2 Genomic-Metabolic Unit, University Marista Of Merida, Yucatan, MEX; 3 General Surgery, Hospital Regional Elvia Carrillo Puerto, Institute for Social Security and Services for State Workers (ISSSTE), Yucatan, MEX; 4 Genomic-Metabolic Unit, University Marista Of Merida, Yucatán, MEX; 5 Research and Development, Hospital Regional Elvia Carrillo Puerto, Institute for Social Security and Services for State Workers (ISSSTE), Yucatan, MEX; 6 Surgery, Hospital General Tacuba, Institute for Social Security and Services for State Workers (ISSSTE), Mexico City, MEX

**Keywords:** biliary ileus, biliary litiasis, bilioenteric fistula, bouveret's syndrome, duodenal obstruction

## Abstract

Bouveret's syndrome is a rare disorder that causes upper gastrointestinal obstruction, typically in elderly patients with a history of chronic cholelithiasis. We present an unusual case of a 58-year-old woman with untreated vesicular lithiasis who developed Bouveret's syndrome. She presented with severe abdominal pain, nausea, vomiting, and abdominal distension. Imaging studies confirmed the presence of an impacted gallstone in the duodenum, causing obstruction. The patient underwent successful surgery with removal of the stone and repair of the bilioenteric fistula. Early diagnosis and prompt surgical intervention are essential to prevent complications such as bowel perforation and sepsis. This case highlights the importance of considering Bouveret's syndrome in the differential diagnosis of bowel obstruction, particularly in elderly patients with a history of cholelithiasis.

## Introduction

Bouveret's syndrome is a rare condition characterized by the passage of a gallstone through a bilioenteric fistula into the duodenum, causing intestinal obstruction. The condition is most commonly observed in elderly women, often due to the high prevalence of chronic cholelithiasis in this population. The clinical presentation usually includes non-specific gastrointestinal symptoms such as abdominal pain, nausea, vomiting, and bloating, which may delay diagnosis. This article presents the case of a 58-year-old woman with a history of untreated cholelithiasis who developed upper gastrointestinal obstruction. A computed tomography scan revealed an impacted stone in the second part of the duodenum, prompting surgical intervention after conservative management failed. We review the diagnostic and therapeutic approach to Bouveret's syndrome, emphasizing the critical role of imaging and surgical intervention in improving patient outcomes.

## Case presentation

A 58-year-old woman with a history of controlled hypertension on losartan 50 mg daily and a diagnosis of gallstone disease 10 years ago presented with progressive colicky abdominal pain, mainly in the right hypochondrium and epigastrium, radiating to the mesogastrium. Despite being advised to undergo cholecystectomy after diagnosis, the patient declined surgery, citing the absence of symptoms or complications in the intervening years. Over the five days prior to admission, the pain had worsened, resulting in almost complete intolerance of oral intake, persistent nausea, anorexia, and mild weight loss. On arrival at the hospital, she was dehydrated, tachycardic at 98 beats per minute, and had signs of abdominal distension. On examination, her abdomen was tense, with pain to deep palpation in the epigastrium and right flank and diminished peristaltic sounds. Initial laboratory results showed mild leukocytosis with neutrophilia, elevated C-reactive protein, hypochloraemia, and hypokalemia, suggesting an inflammatory process involving the upper gastrointestinal tract. Although these findings were suggestive of inflammation, further evaluation was required to rule out other possible diagnoses (Table 1). 

**Table 1:** Comprehensive analysis of initial laboratory findings and reference ranges

Parameter	Result	Reference interval
White blood cells	11,500/mm³	4,000–10,000/mm³
Neutrophils	85%	40–70%
C-reactive protein	12 mg/dL	< 0.5 mg/dL
Serum chloride	96 mmol/L	98–107 mmol/L
Serum potassium	3.2 mmol/L	3.5–5.1 mmol/L
Hemoglobin	12.5 g/dL	12–16 g/dL (female)
Platelet count	250,000/mm³	150,000–400,000/mm³
Sodium	135 mmol/L	135–145 mmol/L
Blood urea nitrogen (BUN)	20 mg/dL	7–20 mg/dL
Serum creatinine	0.8 mg/dL	0.6–1.2 mg/dL
Total bilirubin	1.1 mg/dL	0.1–1.2 mg/dL
Alkaline phosphatase	150 U/L	44–147 U/L
Aspartate aminotransferase (AST)	35 U/L	10–40 U/L
Alanine aminotransferase (ALT)	42 U/L	7–56 U/L

**Figure 1:**
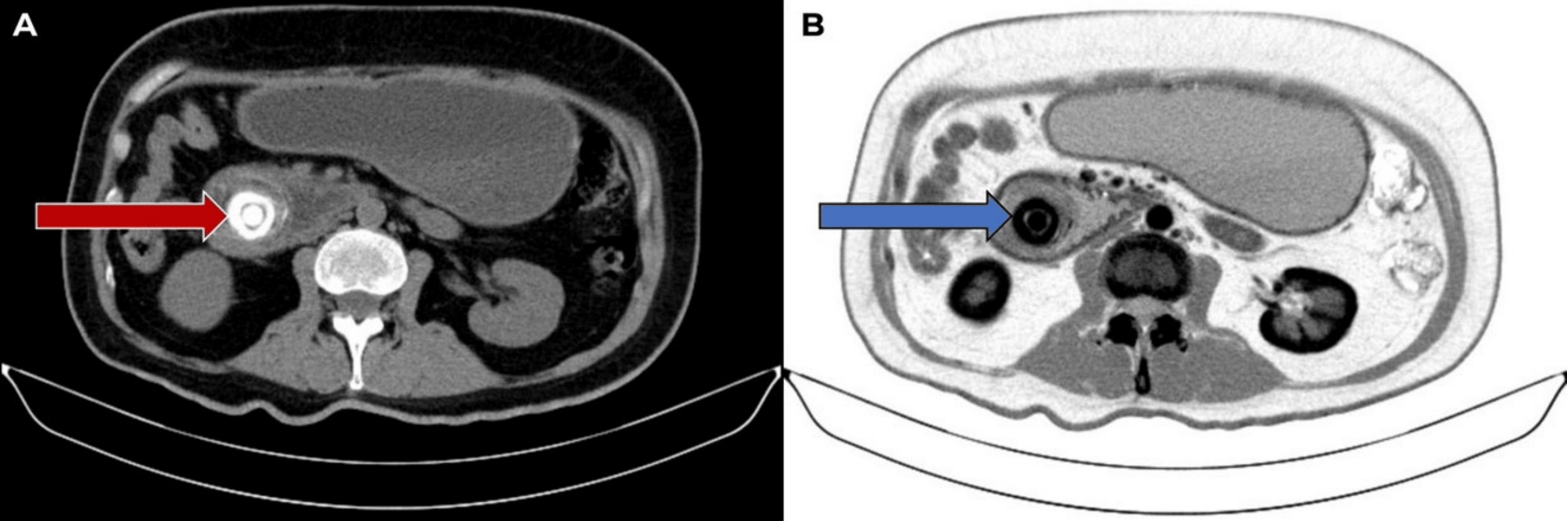
Axial tomography. A) Normal contrast (red arrow), B) Inverted contrast (blue arrow) Axial tomography of the abdomen shows a dilated gastric chamber due to an obstruction in the third part of the duodenum caused by a bile duct. In image A, with normal contrast, the biliary tree can be seen (red arrow), the location of which is confirmed in image B, with inverse contrast (blue arrow), which allows the obstruction to be additionally visualised. These findings are characteristic of Bouveret's syndrome, in which a gallstone impacted in the duodenum obstructs the passage of gastric contents, causing gastric distension and obstructive symptoms in the patient.

Due to the epigastric pain and abdominal distension, a complicated peptic ulcer was initially suspected because of the recurrent self-limiting episodes. Chronic pancreatitis was also considered, given the radiating pain and anorexia, although normal amylase and lipase levels ruled this out. Irritable bowel syndrome was also considered, but the complete oral intolerance and progressive bloating were inconsistent with this diagnosis. In addition, obstructive gastric or duodenal cancer was considered due to chronic pain, weight loss, and oral intolerance. An abdominal ultrasound showed a distended gallbladder with multiple nodules and echogenic material consistent with biliary sludge. The absence of perivascular fluid and the wall thickness (6 mm) were not consistent with acute cholecystitis. The ultrasound also showed significant gastric distension with food debris, suggesting upper GI obstruction. A computed tomography scan confirmed the presence of a 4 cm stone embedded in the second duodenal portion, causing gastric and proximal duodenal obstruction consistent with Bouveret's syndrome (Figure 1).

**Figure 2:**
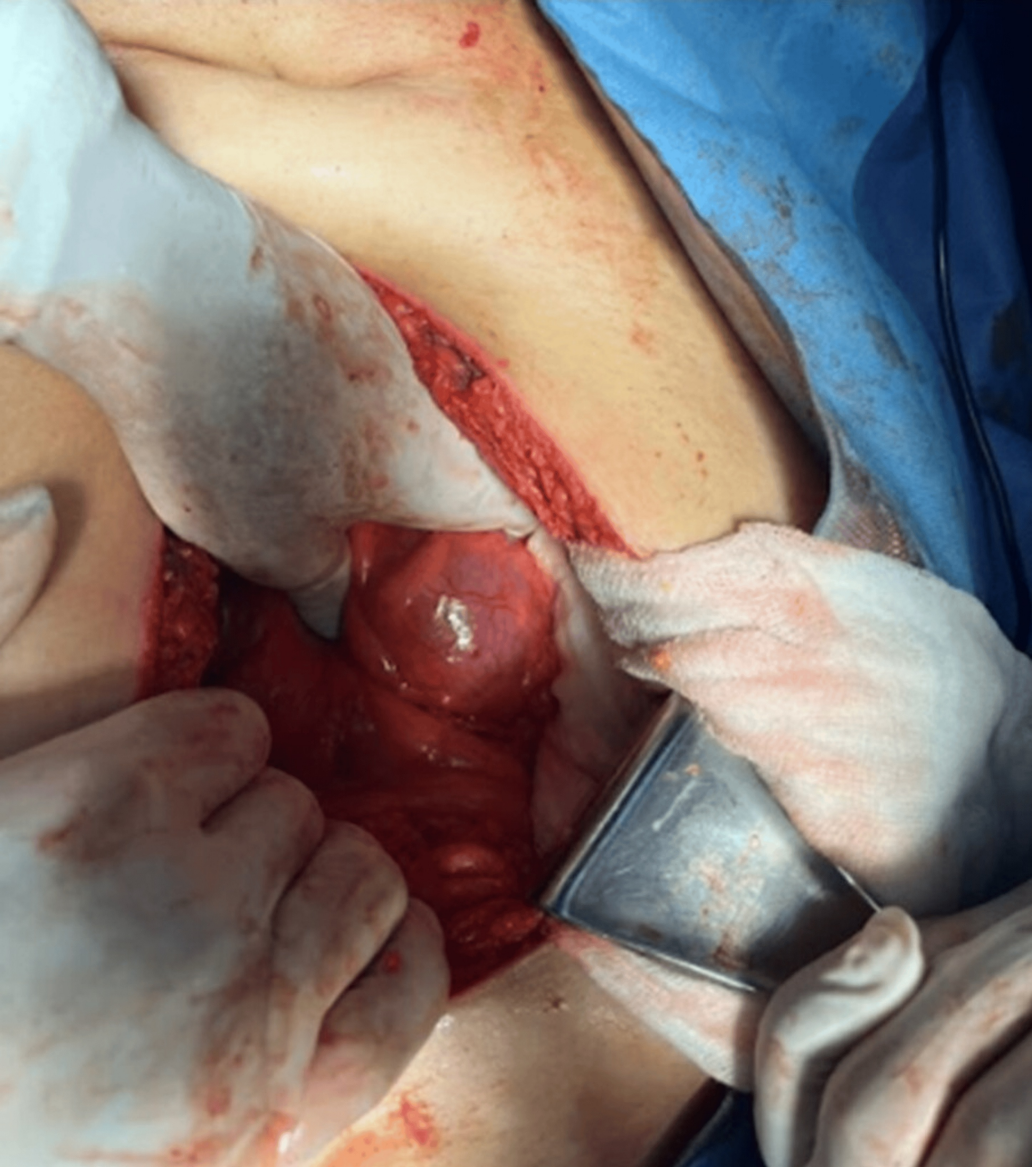
Litho exposure Surgical exposure of an impacted biliary stone at the junction between the second and third portions of the duodenum, performed through a transmesocolic approach. This technique allowed direct access to the stone, facilitating its safe removal. The procedure was necessary due to the obstruction caused by the stone, consistent with Bouveret's syndrome, and allowed preservation of the continuity of the gastrointestinal tract, with minimal manipulation of the surrounding tissues.

**Figure 3:**
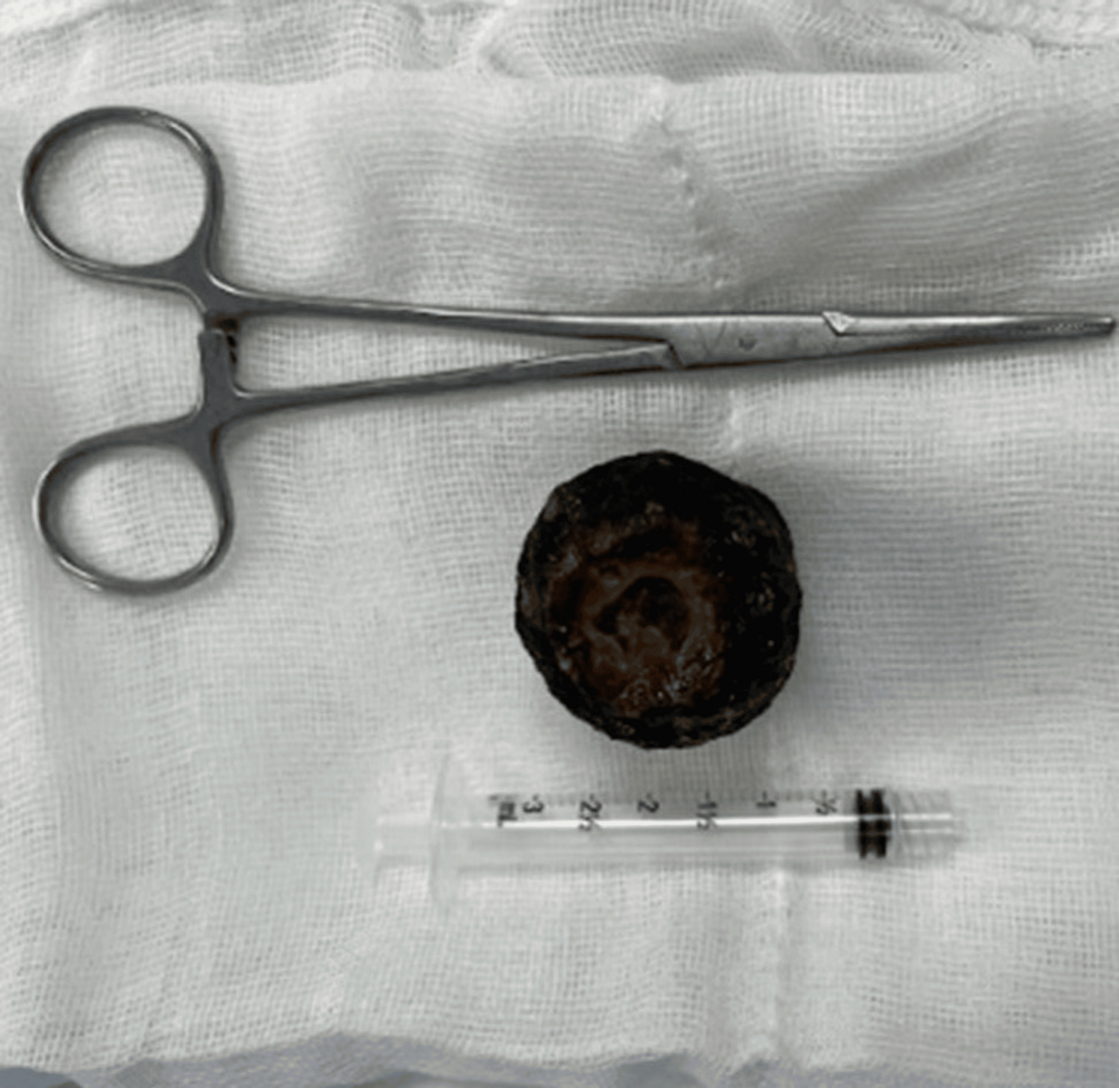
Extracted biliary lithoid of approximately 4 x 4 cm in size A biliary stone measuring approximately 4x4 cm was extracted and impacted in the third part of the duodenum. This stone, which caused the obstruction characteristic of Bouveret's syndrome, was removed during surgery without fragmentation to avoid damage to adjacent structures. The successful removal of the stone relieved the obstruction and restored normal gastric and duodenal transit.

After confirmation of duodenal obstruction due to Bouveret's syndrome, the patient was admitted to the hospital, and a nasogastric tube was placed, draining 1200 ml of gastric and biliary contents in the first 24 hours. Despite this, the patient's abdomen remained distended, and oral intolerance persisted, so an exploratory laparotomy was performed. The procedure revealed significant dilatation of the proximal duodenum, with a 4 cm stone obstructing transit to the jejunum (Figure 2). The gallbladder appeared retracted and adherent due to chronic inflammation, suggesting multiple unresolved episodes of inflammation. Attempts to mobilize the stone into the gastric chamber failed due to its size and anatomical angulation. A duodenal enterotomy was performed via a transmesocolic approach, and the stone was removed without fragmentation to avoid damage to the surrounding tissue (Figure 3). The enterotomy was closed in two layers: the first with 3-0 Vicryl absorbable suture in Connel-Mayo stitches, followed by a reinforcement layer of 3-0 silk in Lembert stitches. The integrity of the repair was checked, and a handmade Saratoga drain was placed to monitor for leaks or postoperative complications. The abdomen was closed in anatomical planes to ensure hemostasis and proper closure.

The immediate postoperative course was uneventful, and the patient was transferred to intensive care for rehydration and electrolyte correction. Her recovery was favorable, and on the third day, she was started on a liquid diet, gradually progressing to a soft diet. The drain was removed on postoperative day six as there was no leakage or infection. She was discharged on day seven in good condition, with recommendations for follow-up in general surgery and gastroenterology. At the one-month follow-up, the patient remained asymptomatic, with good dietary tolerance and no evidence of complications. This case highlights the diagnostic challenges of Bouveret's syndrome, as its symptoms closely mimic more common gastrointestinal disorders such as complicated peptic ulcer, chronic pancreatitis, and gastrointestinal neoplasms. Accurate clinical diagnosis and imaging played a critical role in ensuring timely intervention, highlighting the importance of considering Bouveret's syndrome in patients with a history of biliary lithiasis and vague symptoms.

## Discussion

Bouveret's syndrome is a rare but significant cause of upper gastrointestinal obstruction, particularly in elderly patients with a history of chronic cholelithiasis [1]. The migration of a gallstone through a bilioenteric fistula leads to duodenal obstruction, a condition that often presents with non-specific gastrointestinal symptoms such as abdominal pain, nausea, vomiting, and weight loss [2]. The rarity of this syndrome, combined with its clinical similarities to other more common conditions, can make early diagnosis challenging, often resulting in delayed treatment and increased morbidity [3].

This case illustrates the critical importance of considering Bouveret's syndrome in the differential diagnosis when evaluating elderly patients with a history of cholelithiasis and unexplained abdominal symptoms [4]. The patient's presentation of severe epigastric pain, vomiting, and weight loss initially suggested more common diagnoses such as peptic ulcer disease or chronic pancreatitis. However, advanced imaging techniques, particularly computed tomography (CT), were essential in confirming the diagnosis by clearly identifying the obstructing stone in the duodenum and the associated biliary fistula [5]. CT has proven to be a valuable diagnostic tool in Bouveret's syndrome, with sensitivity rates in excess of 90%, providing not only a clear view of the obstruction but also critical anatomical details to guide surgical planning [6].

Surgical intervention remains the standard of care, particularly in cases with large stones or persistent obstruction, as seen in this patient. The case also highlights the success of a combined approach involving both stone extraction and bilioenteric fistula repair, which can be performed during the same surgical procedure if anatomical conditions allow [7]. Early and appropriate surgical intervention is essential to prevent further complications such as bowel perforation, sepsis, or peritonitis [8].

This case highlights the need for increased awareness among clinicians, particularly in regions with high rates of cholelithiasis, where Bouveret's syndrome may be under-recognized [9]. Given the non-specific nature of its symptoms, a more proactive diagnostic approach - incorporating advanced imaging techniques - may improve early detection, leading to better clinical outcomes [10]. Furthermore, it highlights the importance of multidisciplinary collaboration between surgeons, gastroenterologists, and radiologists to ensure timely and effective management of this rare condition [11].

In conclusion, although Bouveret's syndrome remains a rare diagnosis, it is an important consideration in patients with a history of cholelithiasis presenting with non-specific gastrointestinal symptoms. Early recognition and appropriate intervention are essential to minimize morbidity and ensure favorable outcomes for affected patients [12].

## Conclusions

Bouveret's syndrome is a rare cause of upper gastrointestinal obstruction that should be considered in elderly patients with a history of cholelithiasis who present with non-specific symptoms. Advanced imaging techniques, particularly computed tomography, play a crucial role in confirming the diagnosis and guiding treatment. Surgical intervention, including gallstone removal and fistula repair, remains the cornerstone of management, especially for large stones or persistent obstructions. This case highlights the importance of early recognition and a multidisciplinary approach to improve outcomes and prevent complications associated with this rare condition.

## References

[1] Parvataneni S, Khara HS, Diehl DL (2020). Bouveret syndrome masquerading as a gastric mass-unmasked with endoscopic luminal laser lithotripsy: a case report. World J Clin Cases.

[2] Navarro-Del Río E, Hernández-Zúñiga JF (2020). Bouveret´s syndrome: a rarest complication of cholelithiasis. A case report and literature review. Cir Cir.

[3] Varre JS, Wu JL, Hopmann P, Ruiz O, Reddy R (2021). Endoscopic and surgical management of Bouveret's syndrome complicated by gallstone ileus. J Surg Case Rep.

[4] Bruni SG, Pickup M, Thorpe D (2017). Bouveret's syndrome-a rare form of gallstone ileus causing death: appearance on post-mortem CT and MRI. BJR Case Rep.

[5] Ferhatoğlu MF, Kartal A (2020). Bouveret's syndrome: A case-based review, clinical presentation, diagnostics and treatment approaches. Sisli Etfal Hastan Tip Bul.

[6] Giuffrè M, Macor D, Masutti F (2019). Evaluation of spleen stiffness in healthy volunteers using point shear wave elastography. Ann Hepatol.

[7] Sadovnikov I, Anthony M, Mushtaq R, Khreiss M, Gavini H, Arif-Tiwari H (2021). Role of magnetic resonance imaging in Bouveret's syndrome: a case report with review of the literature. Clin Imaging.

[8] Caldwell KM, Lee SJ, Leggett PL, Bajwa KS, Mehta SS, Shah SK (2018). Bouveret syndrome: current management strategies. Clin Exp Gastroenterol.

[9] Farkas N, Kaur V, Shanmuganandan A, Black J, Redon C, Frampton AE, West N (2018). A systematic review of gallstone sigmoid ileus management. Ann Med Surg (Lond).

[10] Wang F, Du ZQ, Chen YL, Chen TM, Wang Y, Zhou XR (2019). Bouveret syndrome: a case report. World J Clin Cases.

[11] Parra-Pérez V, Watanabe-Yamamoto J, Nago-Nago A (2015). Factors related to advanced colorectal neoplasm at the Policlínico Peruano Japonés. Rev Gastroenterol Mex.

[12] Qasaimeh GR, Bakkar S, Jadallah K (2014). Bouveret's Syndrome: an overlooked diagnosis. A case report and review of literature. Int Surg.

